# Harnessing the Persuasive Power of Narrative: Science, Storytelling, and Movie Censorship, 1930–1968

**DOI:** 10.1017/S0269889718000029

**Published:** 2018-03

**Authors:** David A. Kirby

**Affiliations:** University of Manchester E-mail: david.kirby@manchester.ac.uk

## Abstract

As the deficit model's failure leaves scientists searching for more effective communicative approaches, science communication scholars have begun promoting narrative as a potent persuasive tool. Narratives can help the public make choices by setting out a scientific issue's contexts, establishing the stakes involved, and offering potential solutions. However, employing narrative for persuasion risks embracing the same top-down communication approach underlying deficit model thinking. This essay explores the parallels between movie censorship and the current use of narrative to influence public opinion by examining how the Hays Office and the Catholic Legion of Decency responded to science in movies. I argue that deploying narratives solely as public relations exercises demonstrates the same mistrust of audiences that provided the foundation of movie censorship. But the history of movie censorship reveals the dangers of using narrative to remove the public's agency and to coerce them towards a preferred position rather than fostering their ability to come to their own conclusions.

Discussions amongst policymakers, scientific organizations, and scholars about the nature of science communication have changed dramatically since the dissolution of the Royal Society's Committee on the Public Understanding of Science (COPUS) in 2002. Social scientists blamed COPUS's failure on its reliance on a conceptualization of science's relationship to the public known as the “deficit model” (Miller 2001). The deficit model adopts a one-way, top-down communication process that attributes negative attitudes towards science and technology to the public's lack of information.^1^ This communicative mode assumes that the best way to combat negative attitudes towards contemporary science and technology is for scientists to fill this knowledge vacuum. Significant critiques of the deficit model began appearing in the early 1990s that questioned the foundation and effectiveness of this approach (Hilgartner 1990; Wynne 1995; Evans and Durant 1995). Since 2002, science communicators tried to replace the old focus on the top-down notion of “scientific understanding” with an emphasis on “dialogue” and two-way communication between scientists and non-scientists (Gregory and Lock 2008; Bubela et al. 2009; Nisbet and Scheufele 2009).

The failure of approaches based on science literacy to persuade the public has left many in the scientific community concerned that science is losing out in its fight over controversial areas such as climate change, evolution, biotechnology, and vaccinations. Science communication researchers have proposed a number of alternative techniques for improving the persuasiveness of science communication efforts including an emphasis on deliberative forums, trust building, and framing (Nisbet and Scheufele 2009). Other scholars have promoted the use of narrative and storytelling in the context of entertainment as a powerful means to communicate science in a persuasive fashion (Avraamidou and Osborne 2009; Negrete and Lartigue 2010; Dahlstrom 2014; Kaplan and Dahlstrom 2017; Martinez-Conde and Macknik 2017). Marine biologist turned filmmaker Randy Olson, for example, believes that science communication has a “narrative deficiency” that is preventing the scientific community from effectively communicating with the public (Olson 2015, 8). The general consensus is that scientists have to tell their own stories about science because their opponents are already creating alternative persuasive narratives.

Research in the field of narrative persuasion suggests that entertainment media narratives have a powerful persuasive capacity (Moyer-Gusé and Dale 2017). Narratives are effective in altering attitudes and behaviors because they reduce various forms of resistance to persuasion. The perceived reality of entertainment stories fosters “transportation” into a narrative world, which involves an integrative melding of attention, imagery, and feelings that become focused on story events (Green, Brock, and Kaufman 2004). Audiences that are transported feel removed from their surroundings and completely engaged in the narrative's world (Green and Brock 2000). Transportation can have powerful persuasive consequences because it promotes emotional engagement with the characters and it reduces the ability of audiences to construct counterarguments. Scholars have also argued that in the “post-truth” world narrative plays a significant role in establishing and legitimating “facts” (Howe 2017).

Scientists’ deployment of fictional narratives for persuasive purposes is not a new phenomenon (Milburn 2002; Haran et al. 2007; Mellor 2007). But the scientific community's desire to utilize narrative has increased, as evidence emerges that facts presented on their own do not have the persuasive power that was once assumed. Many high profile scientific organizations – most prominently the US National Academy of Sciences’ Science and Entertainment Exchange and USC's Hollywood Health and Society – have developed initiatives to harness the influence of entertainment narratives in persuading otherwise resistant audiences about issues related to science (Kirby 2017). Climate change has also emerged in the last decade as a dominant theme in literature, film, and theatre (Johns-Putra 2016; Svoboda 2016). The goal of what has been dubbed “cli-fi” is to warn the public about the dangers of climate change by “translating graphs and scientific jargon into experience and emotion” (Tuhus-Dubrow 2013).

Science communicators’ budding embrace of narrative certainly represents a dramatic shift away from approaches based on the unambiguous dissemination of facts. But storytelling approaches are not completely free from the trap of deficit model thinking. Deficit model thinking is not just about information transfer, it also involves ensuring that the public uses this information to make what scientists believe is the “right choice” about scientific issues. Three assumptions form the foundation of deficit model thinking: 1) that there is a single “correct” conclusion for any scientific controversy that affects the public, 2) that given the right amount of appropriate information the public will reach this conclusion and, 3) that communication of uncertainty will undermine public trust in science (Ahteensuu 2012). Deficit model thinking in science communication survives in ways that are both overt and subtle because its assumptions are rooted in a mistrust of the public's ability to make their own decisions.

Even without focusing on science literacy, top-down models still shape the views and actions of scientists and decision makers. Alan Irwin points out that many scientists and scientific organizations still employ phrases such as “managing public opinion” that show that they are more concerned about controlling the ways in which the public thinks about science than they are about engaging in dialogue (Irwin 2014). For Michael Dahlstrom and Shirley Ho the idea of selling science to the public is a potential ethical trap that scientists encounter when they consider using narrative for science communication. They suggest that communicators ask themselves if the underlying purpose of using narrative is for comprehension or persuasion (Dahlstrom and Ho 2012, 592). If a communicator's goal is merely persuasion then narrative becomes just another top-down approach based on deficit model thinking that potentially undercuts other attempts to build trust with the public. Roderick Hart and David Payne also note that narrative in the service of persuasion is “especially seductive . . . because it hides highly directive prescriptions within seemingly neutral descriptions” (Hart and Payne 1990, 374).

Scientists who rely on storytelling approaches for persuasive purposes are trying to remove ambiguity from their messages, which they believe will make it “easier” for the public to come to the “correct” decisions about a scientific issue. But such approaches still reflect deficit model thinking because they are about removing the public from dialogue for their own protection. The same assumptions underlying the deficit model of science communication provided the foundation for movie censorship in the twentieth century. Movie censorship was also a top-down communicative approach that was built upon a mistrust of how audiences interpret messages and make decisions. I believe that we can learn about the dangers and limitations of using narrative as a science communication tool by examining these previous attempts to control fictional narratives including the stories told about science in cinema.

In this essay, I will explore the historic parallels between movie censorship and the continuing role the deficit model plays in the use of narrative as a persuasive science communication technique by examining the ways in which censors in the US responded to science in cinema between 1930 and 1968. The Catholic Church and other Christian organizations such as the Woman's Christian Temperance Union, Federation of Churches, and the National Council of Churches of Christ in America played a central role in the creation and administration of Hollywood's self-censorship organization the Motion Picture Producers and Distributors of America, which was popularly known as the Hays Office, as well as the other major censorship group in the US the Catholic Legion of Decency (Black 1996; Walsh 1996; Leff and Simmons 2001). Hollywood had established the Hays Office in 1922 as a response to protests by these and other religious groups over what they saw as immoral content in movies. By 1930 studio heads agreed to abide by a code of standards called the Motion Picture Production Code written by two prominent Catholics (Leff and Simmons 2001).

Although many scholars refer to the work of the Hays Office and the Legion of Decency as censorship, the goal of these groups was generally not to prevent studios from releasing films. As film scholar Lea Jacobs argues, the Hays Office did not operate through the restraint of exhibition but instead that censorship was about regulating the production of meaning in texts. Jacobs demonstrates that the Hays Office's mode of censorship operated at the level of representation and that it involved a process of “textual determination” by which they could help the studios craft what they felt were more appropriate stories (Jacobs 1989, 4).

After the development of the Production Code, censorship organizations took the approach of closely analyzing, commenting upon, and recommending changes to the content of every story treatment and script in order to control a film's dialogue, visuals, individual scenes, character motivations, plot points, and overall narrative. The censors wanted to make sure that film content was morally appropriate or, at the very least, to make sure the texts were not blasphemous, indecent, or legitimating what they considered to be dangerous ideas. Censors’ concerns about morality were not limited to nonverbal, visual aspects such as sex and violence; they were just as concerned with the portrayal of less tangible elements whose meanings were open to interpretation. This involved representations associated with science including the social, political and cultural aspects of science practice and scientific knowledge.

By exploring how movie censorship groups tried to control the production of meaning in film narratives I hope to help narrative become a more useful technique that science communicators can deploy in an ethical and productive manner especially for salient issues like GMOs, AMR, climate change, and vaccinations. I find that the different censorship groups adopted different perspectives on whether or not to trust audiences to interpret movie narratives in what they considered to be the correct manner. In addition, I show how movies censors believed that science was not a suitable subject matter for the lowbrow mass medium of motion pictures. Science was dangerous for these censorship groups because it provided films with an added level of realism that might confuse audiences about the fantastical nature of the stories and might prevent them from critically assessing a film's messages. Censors also considered science to be far too complicated to be understood by the public without suitable guidance. The censors did not trust the public to interpret the thematic implications of science in cinema and they felt that these misunderstandings could be dangerous. Ultimately, I argue that narratives can provide the public with another tool for making choices by setting out the context for a scientific issue, establishing the stakes involved, providing useful information, and offering potential solutions. But the history of movie censorship shows us the dangers of using narrative to remove the public's agency by coercing them towards a preferred position rather than fostering their ability to make their own choices.

## To Trust the Public or Not? Movie Censorship in the Pre-Code Era

In 1930 the Studio Relations Committee (SRC) was the branch of the Hays Office tasked with enforcing the Production Code. Colonel Jason Joy and his successor James Wingate ran the SRC from the adoption of the Production Code in 1930 until its reformation as the Production Code Administration (PCA) in 1934 (Black 1996; Doherty 1999).^2^ The SRC advised studios on how to alter their scripts so that they met the standards of the Production Code. The SRC, however, could not force studios to accept their suggestions. This meant that despite their agreement to abide by the Production Code, studios frequently ignored the SRC's recommendations in the early 1930s (Olasky 1985; Doherty 1999). In addition, Joy and Wingate took a particularly lenient approach to the Production Code, which they viewed as a flexible set of general guidelines rather than a rigid set of rules (Black 1996). Confusingly, the time period between when the SRC adopted but laxly enforced the Production Code in 1930 and the creation of the PCA in 1934 is referred to as the pre-Code era.

For Joy and Wingate, movie censorship was primarily about protecting audiences from indecent depictions of sex and violence or from overtly immoral stories. They did not believe that the SRC should prevent studios from producing films with controversial topics as long as the films handled these topics in an intelligent fashion. According to film scholar Gregory Black, “Films that were considered immoral by religious clergy and other guardians of morality were often seen by Joy and Wingate as good entertainment, satire, comedy, or legitimate commentary on contemporary social, moral, or political issues” (Black 1996, 53). In essence, they believed that adult audiences could handle thought provoking stories as long as these stories did not blatantly incorporate immoral, offensive, or blasphemous messages. They also felt that films could communicate moral messages if the film's narrative punished characters who supported contentious ideas.

Joy and Wingate's lenient position meant that, in the pre-Code era, studios were often able to use narrative ambiguity to tell some semblance of the stories they wanted to tell about science. Film historian Richard Maltby explains that the Production Code “permitted producers to deny responsibility for a movie's content, through a particular kind of ambiguity, a textual indeterminacy that shifted the responsibility for determining what the movie's content was away from the producers to the individual spectator” (Maltby 2003). Shifting the responsibility of interpretation to the viewers was in line with Joy and Wingate's approach to administrating the Production Code. In fact, Joy recommended that studios develop a system of narrative and representational conventions “from which conclusions might be drawn by the sophisticated mind, but would mean nothing to the unsophisticated and inexperienced” (quoted in ibid.).

Ambiguity became a narrative strategy as studios relied on veiled language and polysemous scenes that tolerated multiple interpretations. This ambiguity prevented censors from removing storylines because they could not prove that they were explicitly about controversial ideas. But, long-standing narrative codes and conventions made it clear to supposedly more sophisticated members of the audience what the stories were about. According to Maltby, this strategy became “an enabling mechanism at the same time that it was a repressive one” by permitting educated audience members to join the conversation while preventing illiterate audience members from being exposed to problematic topics (ibid.).

The SRC's approach to movie censorship during the Pre-Code era was in stark contrast to the religious reformers and the various regional censor boards that were pushing for stronger censorship measures. The SRC referred to these various regional censor boards, including city and state censor boards in the US as well as international censors, as the political censor boards. The difference in approach between the SRC under Joy/Wingate and the political censor boards was significant because films approved by the SRC had to pass through the political censor boards before they could be shown in these regions. The political boards almost uniformly believed that cinema possessed a persuasive power that legitimated controversial concepts including scientific ideas like evolution, eugenics, psychology, and patent medicines. Therefore, they felt that what they considered to be morally problematic ideas should be kept out of movies.

The censorship history of Universal Pictures’ 1931 adaptation of *Frankenstein* is illustrative of how Joy attempted to mitigate the potential negative influence of cinematic stories during the Pre-Code era while also trying to allow studios to produce movies with challenging narratives. The film's original script passed through a lenient Hays Office with little difficulty.^3^ Joy's only suggestion was for the removal of a line of dialogue overtly comparing Frankenstein's act of scientific creation to God, “In the name of God! Now I know what it feels like to be God!” After seeing the final film Joy was certain that it would be “reasonably free from censorship action” by the political censor boards.^4^

Joy's optimistic prediction turned out to be completely wrong. The film ran into significant opposition from the numerous regional and international political censor boards. Political censor boards felt that two factors made the film dangerous for public consumption. One was the horrific nature of the monster and the film's visuals. The other element was the blasphemous nature of the narrative, which involved a scientist usurping God's role by scientifically creating life. “Blasphemy” was the issue that led to censors banning the film in several locations including Quebec and Kansas.^5^ The Kansas censor board's initial rejection of the film makes these objections clear:

The reason is given because of BLASPHEMY which Webster says is . . . the act of claiming the attributes or prerogatives of the deity. Besides being an Ecclesiastical offence, blasphemy is a crime at the common law, as well as generally by statute, as tending to a breach of the peace and being a public nuisance or destructive of the foundations of civil society.^6^

Generally, the filmmakers worked with the censor boards to overcome objections by removing dialogue or by cutting out scenes that were problematic. If the blasphemy in *Frankenstein* were confined to a scene or two then the solution was to simply remove these scenes. But the blasphemous notion of a scientist creating life is central to the entire film. There was no way for the studio to overcome this objection without fundamentally changing the film's narrative, which was an unacceptable solution for both the studio and Joy.

Eventually, the studio satisfied censors’ objections by simply adding a short prologue to the start of the film. This might seem to be a perplexing solution given that the prologue does nothing to change the blasphemous nature of the story. The film's narrative still involved a scientist creating life after the prologue ended. But the political censor's problems were not just with the fictional story. This particular story had existed in book form for over a hundred years. Instead, their anxiety stemmed from their conviction that the persuasive power of an audiovisual medium – cinema – would lead audiences to believe that a scientist could create life in a laboratory. Movie censors feared that uneducated working-class audiences were not capable of realizing that what they were seeing on the movie screen was just a story and that the public could not distinguish fiction from reality. Therefore, the studio suggested the following prologue to remind audiences that the story they were about to see was a fantasy and did not represent the real state of the world: “The story of ‘Frankenstein’ is pure fiction. It delves into the physically impossible and the fantastic. For almost a hundred years the story has furnished diversion and the picture like the story is for entertainment only. Although no moral is intended it shows what might happen to Man if he challenges the unknowable.”

For Universal Pictures, a prologue was an ideal solution to censorship challenges because it required minimal resources and it kept their films mostly intact. For Joy, the prologue was the perfect form of censorship because it allowed studios to maintain the integrity of their entertaining films but it also reduced the persuasive capacity of cinematic narratives by reminding audiences that these were just stories. The success of *Frankenstein*’s prologue led studios to trot out the prospect of adding a prologue as a means of fending off major cuts to films in the pre-Code era. For example, Universal Pictures suggested a prologue as a way to overcome the equally blasphemous story in *Murders in the Rue Morgue* (1932).^7^ But by the mid-1930s the censor boards found prologues to be an unsatisfactory solution. Ultimately, censorship organizations came to the opinion that film narratives were so powerful in shaping audience's conceptions about the world that even if people were told they were watching a story, movie magic would still convince them that these stories were true to life. From their perspective, the danger of motion pictures demanded an approach that prevented what moral reformers considered to be unacceptable stories from even reaching audiences.

The manner in which Joy/Wingate and Joseph Breen responded to the same film, *Island of Lost Souls* (1932), demonstrates their radically different attitude concerning audiences’ ability to handle challenging narratives. Paramount Pictures initially released the film in 1932 when Joy, and then Wingate, ran the Hays Office. The studio also re-released the film in 1941 which meant that they had to re-obtain approval from a much more rigorous PCA run by Breen. The movie was based on the H.G. Wells’ novel *The Island of Dr. Moreau* and the film's script retained the source novel's evolutionary driven narrative (Kirby 2002). Despite the film's explicit evolutionary themes, it had little difficulty passing through the Hays Office in 1932. As with *Frankenstein*, Joy found nothing in the *Island of Lost Souls* script that he believed violated the Production Code except for one blasphemous line of dialogue uttered by Moreau: “Mr. Parker, do you know what it means to feel like God?” For Joy, and his successor Wingate, a controversial idea such as evolution was an appropriate topic for motion pictures as long as the filmmakers handled the topic in a manner that was not propagandistic.^8^ The Hays Office's official approval letter concluded with Wingate telling the studio that the censors “enjoyed this picture thoroughly” and they believed others would enjoy it as well.^9^

Unfortunately for Paramount, the political censor boards did not share Joy and Wingate's enthusiasm for the movie's narrative. Many censor boards found the plot's overt reliance on evolutionary theory to be unacceptable. The film was “rejected *in toto* by fourteen state censor boards” and it was banned in numerous countries.^10^ The British Board of Film Censors, for example, banned the film until 1958 because of religious concerns over its evolutionary story being “unnatural” as well as its vivisection scenes.^11^ While Joy and Wingate felt that audiences could handle such “unnatural” themes, most censor boards felt that the public was best served by not being exposed to these ideas in a compelling narrative.

From the perspective of advocates for censorship the Hay Office's failure to rigorously enforce the Production Code meant that movies were just as problematic as they were before its adoption. In response, Will Hays created the Production Code Administration (PCA) as a way to curtail continuing calls by moral reformers for a governmental censorship organization (Black 1996). Tough-minded Catholic Joseph Breen took over as director of the PCA in 1934. Breen had the power he needed to force studios to alter their scripts to conform to the Production Code's standards or he would withhold the PCA's Seal of Approval (Leff and Simmons 2001). As such, the PCA exerted significant influence over movies scripts including the ways in which those scripts told stories using science.

This meant that when *Island of Lost Souls* was re-released in 1941, a much more vigilant PCA had to judge the appropriateness of the film's story for audiences. The PCA rejected the film outright because of the evolutionary basis of Moreau's experiments. Breen explained to the studio that the underlying evolutionary aspects of the narrative meant that it could not be approved under the Code. In his judgment letter to Paramount, he wrote: “The general unacceptability of this picture is suggested by the blasphemous suggestion of the character, played by Charles Laughton, wherein he presumes to create human beings out of animals.”^12^ Unlike the case of *Frankenstein*, the political censors were not satisfied with a prologue reminding people that the film was just a story.

Essentially the censors wanted the studio to remove any possibility that the audience could interpret this story as being about the nature of human origins. They could not allow a movie narrative where a character, even a clear villain like Moreau, contradicted the creation story in Genesis. In order to obtain the PCA's approval Paramount had to take out every line of dialogue in the film that suggested that Moreau was “creating” humans by evolving them from animals.^13^ The studio assured the PCA that “these cuts eliminate from the picture the suggestions that Moreau considers himself on par with God as a creator, and reduces him to the status of a scientist conducting bio-anthropological experiments.” In the edited film there is no longer any indication that Moreau made the creatures on the island, he is now merely an anthropologist studying their behaviors. In this way, the beast-people merely become another of God's creations. That was a narrative about science that the PCA deemed appropriate for audiences.

Scholars refer to the pre-Code era as the “pre-Code era” because the Hays Office's SRC only loosely enforced the Production Code during this period. The Hays Office did not strictly adhere to the Production Code because the directors at the time, Joy and Wingate, did not believe that the goal of censorship should be to keep filmmakers from telling stories with difficult themes. Instead, they felt that it was better to allow the public to grapple with controversial themes in cinema as long as films did not present biased versions of these themes. But their views were not in line with the majority of moral reformers including the political censor boards who demanded more stringent censorship. They wanted censors to police cinema in such a way that meant that movies only featured what they considered to be narratives that would persuade audiences to behave in a moral fashion. In essence, these moral reformers wanted the censors to take a top-down approach built upon an assumption that the public could not be trusted to make their own decisions about how to behave. This approach to the persuasive power of narrative was more in line with what we now call deficit model thinking.

## Creating Appropriate Stories about Science through Censorship

American censors were not content with merely preventing morally dangerous narratives from influencing what they saw as vulnerable audiences. In fact, many proponents of censorship wanted to harness cinema's persuasive power in order to transmit stories that would have a positive influence on audiences. This was a goal made clear in the Production Code's opening sentence: “If motion pictures present stories that will affect lives for the better, they can become the most powerful force for the improvement of mankind.”^14^ Censorship for the PCA was not about creating movie narratives that matched our own reality; it was about using cinematic stories to depict an idealized reality that represented what the censors believed should constitute the real world. Therefore, the PCA tried to coerce studios into producing what censors considered to be suitably positive and morally uplifting narratives including those about science.

The PCA's desire for only virtuous stories was a problem for studios that wanted to tell provocative stories about science or stories highlighting potential ethical issues in science. As Susan Lederer notes, the PCA's approach to narratives often prevented mainstream filmmakers from addressing pressing moral, social, and political themes involving science and medicine (Lederer 1998). For the PCA, however, cinema was not a suitable medium by which to have ethical conversations about science or scientific ideas. They did not want movies to pose moral dilemmas about science. According to the PCA, movies were a tool for teaching people right from wrong; and for them right and wrong were clearly discernible. No grey areas were allowed. This stance forced studios to perform narrative gymnastics in order to tell some semblance of the stories they wanted to tell about science's ethical grey areas or about science's role in society.

I will use the PCA's negotiations with Warner Bros. over the script of their 1938 film *The Amazing Doctor Clitterhouse* as a way of demonstrating how the PCA compelled studios to modify their narratives and how studios tried to maintain their stories in the face of these efforts. The story in the original script involved the prestigious scientist Dr. Clitterhouse deciding that the best way to stop crime is to understand the criminal mind and that the best way to understand the criminal mind was to become a criminal himself. At the heart of the script's narrative is the ethical dilemma of whether or not the value of Clitterhouse's scientific findings justified his illegal actions.

After examining this script, the PCA informed Warner Bros. that their story was unacceptable under the principles of the Production Code by allowing that scientific progress justified immoral behaviour:

What primarily concerns us is the general flavor which may be introduced in a motion picture version of this story which would tend to characterize Clitterhouse as a scientific hero, and hence tend to glorify and justify a criminal and a crime on the ground that the purposes of science are served.^15^

The PCA felt uncomfortable with the story of a scientist whose scientific goal is certainly admirable – reducing crime – but who needed to commit crimes in order to accomplish that objective. They told the studio that the only way they would approve this story was if the script overtly depicted Clitterhouse as insane. If Clitterhouse was insane then there was no dilemma about the moral cost of scientific progress. The audience would understand that his actions were obviously wrong because they were the actions of a deranged mind. That was a much more appropriate narrative as far as the PCA was concerned.

Changing the story in this way was problematic for Warner Bros. because they felt that the appeal of the story was its ambiguity. The studio wanted audiences to contemplate the ethical question of whether immoral actions could justify scientific progress. In response to the PCA's letter they changed the script but they also tried to retain the narrative's vagueness about the ethics of Clitterhouse's methods. In the revised script Clitterhouse acts overtly insane but at the end of the film he knowingly winks at the camera to indicate that he is not actually insane. This level of ambiguity about the acceptability of Clitterhouse's actions was still unacceptable to the PCA. They rejected the revised script saying, “In our opinion, this picture is not acceptable from the point of view of the Production Code, for the reason that the ‘question of right and wrong’ was left in doubt.”^16^ From their perspective, there was right and there was wrong; and Clitterhouse was clearly wrong. If the character did not understand that he was wrong then he was clearly insane. They told the studios to remove any ambiguity from the narrative by having the judge officially declare Clitterhouse to be insane.

But this change was unacceptable to Warner Bros. They did not want to tell such a straightforward narrative in which criminal activities are never acceptable even if they might lead to a greater good. The studio still wanted to tell a story in which audiences are left questioning whether Clitterhouse's scientific work justified his crimes. To maintain this uncertainty they changed the ending of the story entirely.^17^ The final script explicitly incorporates the ethical question into the criminal trial by making the jury vote on Clitterhouse's sanity. Ultimately, the jury is split in their opinion of whether Clitterhouse is sane or insane. The ambiguity of the jury's deliberations on this question left open the question of whether or not the ends justify the means for scientific progress, which was the narrative the studio wanted told.

Despite the jury's inability to come to a consensus about the ethicality of Clitterhouse's methods the studio did satisfy the PCA by having the jury ultimately judge him to be insane. But their decision about his sanity was not based on the scientist's belief that committing crimes was an appropriate way to accomplish his scientific goals. Instead the studio preserved their thought-provoking narrative by having the jury pronounce Clitterhouse insane for a completely different reason. In the film the jury argues that while it is in Clitterhouse's best interest to claim that he is insane in order to avoid jail, Clitterhouse continually insists that he and his scientific work are completely sane. From their perspective Clitterhouse's insistence on his sanity proves that he must be insane because only an insane man would work against his own self-interest.

This verdict meant that the studio technically met the censors’ demand. Clitterhouse was found to be insane in a court of law. But the narrative did not provide a definitive answer to the film's ethical question. It was still uncertain as to whether his actions in the pursuit of knowledge were right or wrong. The PCA, however, was satisfied with this ending. From their perspective it was not an ambiguous ending. As with the deficit model, the PCA wanted to live in a black and white world with single answers for complex questions.

## The Authority of Science and Persuasive Narratives

Censorship organizations’ concerns about the persuasive power of mass media were motivated by the fact that moving pictures had a level of realism that had never before been encountered in previous media forms. Unlike literature, proponents of movie censorship considered cinema to be a medium of the masses. Cinema's visuality meant that audiences did not have to be literate to watch a film and censors believed that illiterate audiences did not possess the mental faculties necessary to critically evaluate a film's narrative (Walsh 1996). Those pushing for stronger censorship even feared film adaptations of books that had been around for decades, such as *Frankenstein* (1818) and *The Island of Dr. Moreau* (1895), because they were convinced that cinema's realism would lead illiterate audiences to accept what they were seeing on screen as true including the novels’ problematic messages about the power of science.

Film texts create a reality effect because they are constructed in ways that encourage audiences to immerse themselves in the world or to submit to the pleasures of fiction (Sohn-Rethel 2013, 7). From censors’ perspective, cinema's reality effect was leading uneducated working class and immigrant audiences to forget that what they were seeing on movie screens were just stories, rendering them more susceptible to a film's themes. Film historian Lee Grieveson shows in his study of early movie censorship that the “links between ‘realism’ and ‘suggestibility’ became increasingly central to conceptions of how moving pictures affected audiences” (Grieveson 2004, 64). Groups pushing for formalized movie censorship were worried that cinema's realism was normalizing and legitimating messages transmitted through movies. For moral reformers cinema's reality effect rendered it an extremely powerful and potentially dangerous medium for mass communication.

Sociological studies of cinema in the 1930s such as the Payne Fund studies seemingly provided convincing scientific evidence supporting censors’ assumptions about cinema's persuasive power. The Payne Fund studies – developed by the religiously affiliated Motion Picture Research Council – were one of the first large-scale studies of media effects and they appeared to demonstrate that individual movies could have a strong effect on behavior (Jowett, Jarvie, and Fuller 1996). These influential studies were crucial in the formation of the PCA because they confirmed for proponents of movie censorship that cinematic narratives had a potentially dangerous persuasive power. The Payne Fund studies made the censors’ practice of controlling narratives by closely analyzing and commenting upon the content in every script seem to be the appropriate tactic. If individual films could have such a powerful effect on audiences, then it made sense for these censorship organizations to act as gatekeepers who permitted what they considered to be acceptable narratives to reach the screen and, thus, mass audiences.

Concerns about fictional realism made science particularly problematic for movie censors. Censors considered science dangerous because it provided fictional narratives with an added level of realism that could mislead audiences into accepting problematic stories as legitimate representations of the world. One of the reasons the deficit model has such a continuing appeal for the scientific community is that science has been the Western world's accepted mode of determining truth since the Age of Enlightenment (Psillos 2005). It is easy to see how science's primacy in our culture can lead scientists to believe that the public should uncritically listen to what they have to say.

The notion that science represents objective reality is also one of the major reasons why censor groups wanted to control how science was used in movies. Filmmakers have historically utilized science to enhance the realism of their films and to make cinematic stories seem more plausible (Kirby 2011). For movie censorship organizations, science's ability to make cinematic stories seem more plausible made it a potential moral danger if the censors did not like the stories these movies were telling. Censors were concerned about filmmakers using science to legitimate problematic concepts whether the science was based on real-world scientific concepts or the science was totally fictional. Even fictional science benefits from science's cultural authority as the arbiter of truth.

Censors reacted negatively to scripts that employed science's authority to support what they considered to be philosophically problematic ideas such as spiritualism, secular humanism, and the transmigration of souls. Although the censors disliked these spiritual belief systems, they could not prohibit their inclusion in movies because the ideas did not violate any aspect of the Production Code. However, if a script used scientific explanations –even totally fictional science – to “prove” that one of these concepts was legitimate then it was in violation of the Production Code because the censors believed that such scientific validation in a movie would offend Christian viewers.

In Catholic theology, for example, there is a very clear explanation for what happens to a person's soul after they die: the soul leaves the body, it is judged by God and it is either brought into heaven, sent to purgatory, or condemned to hell. Any film narratives positing alternative beliefs about the nature of the human soul were problematic for the PCA but they could not force studios to alter these narratives just because they did not like these ideas. If the script had characters proffering scientific explanations to legitimate the alternative beliefs about the nature of the human soul, however, then the PCA asked the studio to remove these scientific explanations. The PCA forced studios to eliminate scientific explanations for soul dualism in *Bewitched* (1945), souls put into animals in *Mesa of Lost Women* (1953), and reincarnation in *I've Lived Before* (1956). As far as the PCA was concerned, filmmakers who used science to enhance the legitimacy of a fictional narrative were being dishonest by appropriating the authority of science to support an idea that might not stand on its own terms.

## The Dangers of Real-World Science in a Fictional Narrative

Although fictional science could be an issue, the PCA was even more concerned about fictional narratives that deployed real-world science to validate what they saw as problematic concepts. I will examine the PCA's response to the use of real-world science in the original script for *The Gamma People* (1956) as a way to illustrate their concerns. The film was a typical science fiction B-movie from the mid-50s with a plot about a mad scientist bombarding children with gamma rays in order to transform them into super geniuses. The PCA approved most of the initial script except for one element that made the whole narrative unacceptable. The script included text from Bertrand Russell's 1931 book *The Scientific Outlook* that was to be projected on the screen as an epilogue:

So far, no experiments have been made to test the effect of X-rays on the human embryo. . . . . Sooner or later, however, such experiments will be made. If science continues to advance as fast as it has done recently, we may hope to discover ways of beneficially influencing the human embryo.^18^

The studio's intention was to use Russell's writings as a way to heighten the plausibility of the fictional scientist's experiments. As the PCA's response shows, however, the studio's use of real-world science to legitimate their fictional scenario did not sit well with the PCA:

The appendix superimposed over the closing sequence of this picture would seem to add a note of reality to what we assume to be a story of improbability and would therefore, we feel, render an otherwise acceptable story *un*acceptable under the provisions of the Production Code. We are concerned here with the fact that a well-known scientist apparently has afforded his commendation to the work of a madman.^19^

Although this fictional scientist's experiment was disturbing on its own, the PCA considered it ridiculous enough not to be disturbing for audiences. But the suggestion that the film's gamma ray experiments were grounded in real-life science turned the story from silly to horrifying in their minds. From the censors’ perspective, scientific legitimacy was the real horror.

The essence of the deficit model in science communication is a conviction that science is far too complicated for the public to understand without the help of scientists. Movie censors shared this belief about the nature of science. They felt that there were certain scientific concepts that should never be disseminated through fictional movies because any misunderstandings of these ideas by the audience were morally problematic. They believed that the inclusion of scientific themes in films required expert intercession by real-life scientists or these themes became dangerous. For censors, the simplistic nature of movie narratives meant that the medium was incapable of providing this expert support.

The Legion of Decency's handling of the 1962 bio-picture *Freud* provides a good example of how censors worried about scientific discussions in fictional narratives without appropriate guidance for the audience and it also shows their own deference to the scientific community. Psychiatry was of particular concern for both the PCA and the Legion of Decency because it was the science of human behavior. They believed that misunderstandings of psychiatric theories would lead audiences to believe that science justified certain morally problematic behaviors. For censors, this made the psychiatric sciences a risky topic for movies and they carefully vetted scripts that included psychiatric concepts.

The same pressure from moral reformers that resulted in the creation of the PCA also led the Catholic Church to form the Catholic Legion of Decency in 1933 (Walsh 1996). The Legion of Decency's primary means of censorship was through their film classification system. The Legion's classification system was a guide for Catholic viewers as to what were morally suitable and what were morally questionable films for consumption. They had three levels of classification: A, morally acceptable (with subcategories from I to IV); B, morally objectionable in part; and C, condemned (ibid., 135). Studios were anxious about receiving a B or C classification for their films because they believed that these classifications could seriously impact a film's box office if it drove significant numbers of Catholics away from cinemas (Black 1996, 222). Therefore, studios often negotiated with this censorship group to avoid a B or C classification. This included sending their scripts or their final films to the Legion for approval or recommendations for cuts.

The sexualized nature of cinema was a primary concern for movie censorship organizations. Universal-International Pictures was aware that, for the public, Freud's scientific theories were synonymous with sex. The film's producer William Gordon understood that maneuvering the studio's proposed Freud bio-pic past the censors was going to be difficult and that the “subject matter requires very special, delicate treatment and handling from its inception.”^20^ Therefore, Gordon sent the initial script to the Legion of Decency as a way to preempt any censorship problems before the studio began production. The Legion then asked a number of priests to review the script and offer their opinions of its suitability for the screen.^21^ The priests who reviewed the script agreed that the narrative was not sensationalized. They also felt that the story treated Freud's development of his theories about human sexuality in a restrained and sensitive fashion. They decided that any reasonably intelligent person watching the film would be able to understand these scientific theories without coming to the conclusion that they were all about sex.

The priests’ problem with the script, however, was the fact that they did not believe that most moviegoers were reasonably intelligent people. Father Ray's sentiments were typical of their attitude towards public audiences, “[The script] is suitable for a specialised audience but hardly for unlettered adults, who would tend to dwell on the more sensational aspects of the exposition.”^22^ From the priests’ perspective, the average viewer would not be able to comprehend Freud's complex scientific ideas and, thus, they would misinterpret the narrative and the moral implications of his science. The Legion of Decency makes it clear that such misunderstandings would be “dangerous.”^23^

The Legion's priests did not think that the general public would be able to understand Freud's theories and their moral implications without the assistance from an expert guide like a psychiatrist or a priest. Catholic theologian and literary critic Reverend William F. Lynch made it clear in his review of the script that these ideas required extensive consultation with a trained psychiatrist, not two hours in a movie theatre:

These materials are usually explored under the protection and safeguards of the professional analytic situation, and they take a long, careful time to explore. Doing the same thing in a brief powerful movie is open to the same charge that Dr. Freud brings against Dr. Brener in the text of the ms.: the truth is powerfully given without sufficient help.^24^

In fact, Lynch conceded that even priests might not possess enough expertise to determine the suitability of the science in the script by claiming, “I do not believe it is a question to be resolved by the clergy.”^25^ Instead he recommended that the Legion of Decency only endorse the script if they had assurances from the American Psychiatric Society and the American Psychiatric Association that the science in the script was safe for presentation in the powerfully persuasive medium of cinema. Ultimately, the Legion of Decency deferred to traditional deficit model thinking about science by allowing the scientific community to determine the most appropriate messages about Freudian psychiatry.

Censors felt that the use of real-world science within cinematic narratives was morally dangerous if these narratives led non-experts to embrace immoral behaviors. But movie censorship organizations also worried that scientific knowledge could pose a genuine physical danger if put in the hands of non-experts. The PCA's handling of Marathon Picture Corporation's script titled “The Quiet Murder” usefully illustrates their concerns about fictional narratives depicting non-experts gaining access to powerful scientific information. The PCA rejected every script the studio sent them because the story hinged upon a layperson acquiring specialized scientific knowledge about hypnotism that he then misuses to command a hypnotized woman to commit a murder.

The PCA made it clear in their final decision letter that they would never allow this story to be produced because they wanted to prevent “the inexpert and bizarre use of this science.”^26^ From the PCA's perspective hypnotism was a powerful scientific tool and only experts could be trusted to use this tool appropriately:

The problem with a story like *The Quiet Murder*, seems to lie in the fact that it is perilously likely to awaken a morbid and dangerous curiosity on the subject of hypnotism, and particularly in the criminal potentials of such a power. Hypnotism is a subject which cannot tolerate irresponsible inquisitiveness. It is a field for experts which is likely to be dangerously hurtful to the inexpert.

Consequently, they did not want movies to demonstrate to the inexpert public how to gain this knowledge.

One simple solution for the studio would have been to make the main character an expert in the field of psychiatry rather than an amateur pursuing the science. Marathon Pictures was unwilling to make that change because their goal was to use the film as a warning about the dangers of amateur hypnotism schools. The studio assured the PCA that they would “write a shooting script and produce a picture which will effectively serve to disclose the dangers of hypnotism schools and the evils caused by indiscriminate use of hypnotism in the hands of laymen.”^27^ Their hope was that their film would motivate the public to support legislation such as the Desmond Bill that would “outlaw the use of hypnosis by anyone but an M.D.”^28^

Essentially the studio was caught in a conundrum. In order to establish regulatory legislation, they needed to demonstrate to a mass audience the dangers of non-experts gaining specialist knowledge. But by showing how non-experts could acquire this knowledge they were potentially inspiring lay audiences to imitate what they saw on the screen. The studio's final tactic to obtain approval for their story was appeal to the PCA's shared concerns about the dangers of amateur hypnotism schools.^29^ But their argument that their film represented the greater good despite any potential imitators in the audience was unpersuasive.^30^ No matter how much the PCA wanted to help shut down amateur hypnotist schools, they were more worried about the power of movies to inspire the public to obtain dangerous scientific knowledge that could only be properly applied by scientific experts. That is why the PCA never allowed “The Quiet Murder” to be produced.

## Conclusions: Narrative and Science Communication in a Post-Truth World

The administrators of the Hays Office and the Legion of Decency were not trying to destroy the movie industry. Rather, their goal was to use the persuasive power of cinematic narratives to promote change for a healthier society. They strongly believed that they were acting in a parental role by protecting audiences from inappropriate messages and by helping craft narratives that would have a positive influence on public behavior. Because these groups perceived of movies as a battleground over science's impact on morality, they wanted to regulate how stories about the implications of scientific thought were told in movies. Movie censorship essentially involved these elitist groups deciding for all audiences which narrative interpretations of science and scientific knowledge were suitable for the realist medium of cinema.

There were significant differences amongst these censorship groups as to how science in movies should be censored. The directors of the SRC in the pre-Code era felt that contentious scientific topics were only dangerous if the narratives were constructed in what they felt to be a biased fashion. But their handling of cinematic narratives about science was not in line with moral reformers such as those at the political censor boards who demanded more stringent censorship. The SRC's handling of scientific themes in films like *Frankenstein* and *The Island of Lost Souls* was one factor that led to the creation of the PCA. The PCA believed that the danger of motion pictures demanded an approach that prevented what they considered to be unacceptable stories from even reaching audiences. The PCA demanded control over a film's narrative because they wanted control over how these narratives conveyed the value, implications, and meaning of subjects including science. Essentially, the PCA and the Legion of Decency believed in taking a top-down approach to narrative as a tool for communication.

Now that scientific facts are contested by “alternative facts” in a post-truth world, the need to assess how audiences perceive and interpret scientific narratives is more relevant than ever. It is not difficult to uncover recent examples of fictional narratives whose overt aims are to persuade the public to take a particular side of a scientific controversy. Naomi Oreskes and Erik Conway, for example, followed up their non-fiction polemic *Merchants of Doubt* (2010) with a science-based fictional novel *The Collapse of Western Civilization: A View from the Future* (2014) whose goal is to change public opinion on climate change. Best-selling author Michael Crichton made a career out of using fictional narratives to shape public perceptions of science as an institution and for specific scientific issues including climate change, genetic engineering, nanotechnology, and human/computer interfaces (Radin 2015). Crichton believed that his narrative gained legitimacy because he embedded scientific facts. But in actuality the narratives are what validated the facts in his novels. In order to be successful, fictional texts must be constructed in ways that convince us that they are vehicles of truth.

Narrative's ability to create its own version of truth is what makes it attractive to scientists looking for alternative ways to influence public opinion in light of the traditional deficit model's failure to sway the public. As the case of movie censorship demonstrates, however, using narrative for persuasive purposes runs the risk of embracing a top-down communication approach that is the foundation of deficit model thinking. The use of narratives for science communication should not be built on the elitist assumption that the public needs to be protected from themselves that formed the basis for justifying movie censorship. Movie censors tried to control narratives because they lacked trust in the public to make the right decisions. By creating narratives that feature uncomplicated answers to difficult questions scientists who utilize narratives solely as public relations exercises show this same lack of trust in the public. But this conviction that unambiguous narratives will always change audience behavior highlights one of the principle failings of film censorship and of deficit model thinking in general. Movie censors based their entire approach on the faulty assumption that they could remove ambiguity and complexity from narratives. Movie censorship failed because interpretation is always a part of the movie-going experience. In the end audiences need to decide for themselves what scientific narratives mean to them.

## Footnotes

1 The deficit model is also known as the “diffusionist” model of scientific knowledge (Cooter and Pumfrey 1994).

2 Both the SRC and the PCA were referred to as the Hays Office. However, I will use the designations for the SRC and PCA to differentiate between the Pre-Code Hays Office and the Hays Office after the creation of the PCA in July 1934.

3 (Letter from Joy to Laemmle, 18 August 1931, *Frankenstein* file, PCA archive).

4 Letter from Beeston to Laemmle, 2 November 1931, *Frankenstein* file, PCA archive.

5 Telegram from Leduc to Fithian, 12 January 1932, *Frankenstein* file, PCA archive.

6 Censorship Decision on *Frankenstein*, 17 December 1931, Kansas State Board of Review. Kansas State Historical Society, Kansas State archives.

7 Memorandum from Wilson, 27 February 1932, *Murders in the Rue Morgue* file. PCA archive.

8 Joy's tenure as head of the Hays Office ended before final judgments on the film were made.

9 Letter from Wingate to Hurley, 8 December 1932, *Murders in the Rue Morgue* files. PCA archive.

10 Letter from Breen to Hammell, 18 September 1935, *Island of Lost Souls* file. PCA archive.

11 The UK's censorship of *Island of Lost Souls* is discussed in Skal (1993, 171) and Myers (2012).

12 Letter from Breen to Luraschi, 4 March 1941, *Island of Lost Souls* file. PCA archive.

13 This and subsequent quotation taken from letter from Luraschi to Breen, 15 March 1941, *Island of Lost Souls* file. PCA archive.

14 The Motion Picture Production Code of 1930 can be found in Miller 2012.

15 Letter from Breen to Warner Brothers studios, 12 September 1936, *The Amazing Dr. Clitterhouse* files, PCA archive.

16 Letter from Breen to Warner Brothers studios, 23 April 1938, *The Amazing Dr. Clitterhouse* files, PCA archive.

17 Script pages, 12 March 1938, *The Amazing Dr. Clitterhouse* files, Warner Brothers archive.

18 Louis Pollock, script treatment, undated, *The Gamma People* file, PCA archive.

19 Letter from Breen to Allen, 20 September 1951, *The Gamma People* file, PCA archive (emphasis in original).

20 Letter from Gordon to Little, 28 April 1961, *Freud* files, MPAA archive.

21 Copies of the priests’ opinions on the script are available at the Legion of Decency archive.

22 Letter from Ray to Little, undated, *Freud* files, Legion of Decency archive.

23 A few of the responses from the clergy explicitly use the word “dangerously.” For example, see letter from Cicklic to Gordon, 5 May 1961, *Freud* files, Legion of Decency archive.

24 Lynch, comments on *Freud* script, undated, *Freud* files, Legion of Decency archive.

25 Lynch, comments on *Freud* script, undated, *Freud* files, Legion of Decency archive.

26 This and subsequent quotations are from a Memorandum, 21 February 1949, “The Quiet Murder” files, PCA archive.

27 Letter from Jackson to Baumfeld, 4 July 1948, “The Quiet Murder” files, PCA archive.

28 Letter from Baumfeld to Breen, 24 August 1948, “The Quiet Murder” files, PCA archive.

29 Letter from Satenstein to Breen, 11 February 1949, “The Quiet Murder” files, PCA archive.

30 Letter from Breen to Satenstein, 28 February 1949, “The Quiet Murder” files, PCA archive.
